# Performance of Radiomics Features in the Quantification of Idiopathic Pulmonary Fibrosis from HRCT

**DOI:** 10.3390/diagnostics10050306

**Published:** 2020-05-15

**Authors:** Alessandro Stefano, Mauro Gioè, Giorgio Russo, Stefano Palmucci, Sebastiano Emanuele Torrisi, Samuel Bignardi, Antonio Basile, Albert Comelli, Viviana Benfante, Gianluca Sambataro, Daniele Falsaperla, Alfredo Gaetano Torcitto, Massimo Attanasio, Anthony Yezzi, Carlo Vancheri

**Affiliations:** 1 Institute of Molecular Bioimaging and Physiology, National Research Council (IBFM-CNR), 90015 Cefalù, Italy; alessandro.stefano@ibfm.cnr.it (A.S.); acomelli@fondazionerimed.com (A.C.); viviana.benfante@libero.it (V.B.); 2 Department of Economics, Business, and Statistics (DSEAS), University of Palermo, 90133 Palermo, Italy; maurogioe91@libero.it (M.G.); massimo.attanasio@unipa.it (M.A.); 3 Department of Medical Surgical Sciences and Advanced Technologies, Radiology Unit I, University Hospital “Policlinico-Vittorio Emanuele”, 95123 Catania, Italy; spalmucci@sirm.org (S.P.); basile.antonello73@gmail.com (A.B.); dottorsambataro@gmail.com (G.S.); danielefalsaperla@gmail.com (D.F.); alfre.84@katamail.com (A.G.T.); 4 Regional Referral Centre for Rare Lung Diseases, A.O.U. Policlinico-Vittorio Emanuele, University of Catania, 95123 Catania, Italy; torrisiseby@hotmail.it (S.E.T.); vancheri@unict.it (C.V.); 5 Laboratory of Computational Computer Vision (LCCV), School of Electrical and Computer Engineering, Georgia Institute of Technology, Atlanta, GA 30332, USA; samuel.bignardi@gatech.edu (S.B.); ayezzi@ece.gatech.edu (A.Y.); 6 Ri.Med Foundation, 90133 Palermo, Italy; 7 Artroreuma S.R.L., Rheumatology Outpatient Clinic Associated with the National Health System, 95030 Mascalucia (Catania), Italy

**Keywords:** idiopathic pulmonary fibrosis, high resolution computed tomography, radiomics

## Abstract

Background: Our study assesses the diagnostic value of different features extracted from high resolution computed tomography (HRCT) images of patients with idiopathic pulmonary fibrosis. These features are investigated over a range of HRCT lung volume measurements (in Hounsfield Units) for which no prior study has yet been published. In particular, we provide a comparison of their diagnostic value at different Hounsfield Unit (HU) thresholds, including corresponding pulmonary functional tests. Methods: We consider thirty-two patients retrospectively for whom both HRCT examinations and spirometry tests were available. First, we analyse the HRCT histogram to extract quantitative lung fibrosis features. Next, we evaluate the relationship between pulmonary function and the HRCT features at selected HU thresholds, namely −200 HU, 0 HU, and +200 HU. We model the relationship using a Poisson approximation to identify the measure with the highest log-likelihood. Results: Our Poisson models reveal no difference at the −200 and 0 HU thresholds. However, inferential conclusions change at the +200 HU threshold. Among the HRCT features considered, the percentage of normally attenuated lung at −200 HU shows the most significant diagnostic utility. Conclusions: The percentage of normally attenuated lung can be used together with qualitative HRCT assessment and pulmonary function tests to enhance the idiopathic pulmonary fibrosis (IPF) diagnostic process.

## 1. Introduction

**Figure 1:**
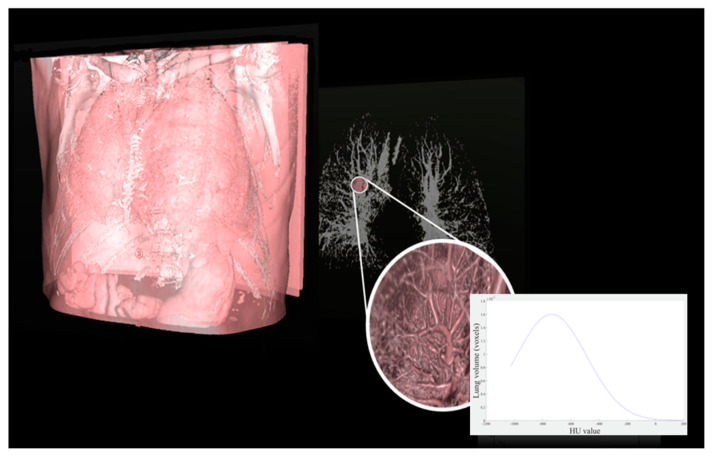
Representative high-resolution computed tomography (HRCT) study and extraction of lung histogram to obtain quantitative parameters.

Idiopathic pulmonary fibrosis (IPF) is a chronic disease characterized by an irreversible decline in lung function [1,2,3]. The progressive development of fibrotic areas within the parenchyma of the lungs is also accompanied by a decline in the patient’s ability to perform everyday activities and, consequently, an overall decline in quality of life [4]. In general, the expected survival time of a patient diagnosed with IPF is typically 3–5 years, which may be stretched to 5–7 years with proper anti-fibrotic therapy [5]. Common parameters for the evaluation of IPF severity include diffusion capacity for carbon monoxide (DLco) and forced vital capacity (FVC), whose variation over time is associated with patient mortality. IPF produces rather distinctive features in high resolution computed tomography imaging (HRCT). For example, traction bronchiectasis and the extent of fibrosis, both obtained from HRCT images, have been reported to be powerful prognostic predictors for mortality. As such, HRCT constitutes a valuable diagnostic tool [5] capable of reducing diagnosis time and avoiding more invasive surgical investigation. Qualitative evaluation of disease extension from HRCT, in combination with physiological parameters, can be used to stage the disease [6]. Unfortunately, the outcome of such visual analysis can vary widely, even when performed by equally trained radiologists. In contrast, it has been demonstrated [7,8,9,10,11] that physiological parameters (e.g., FVC and DLco) closely correlate with parameters derived from HRCT. As such, quantification of the latter has become an important routine practice for IPF assessment and for the detection of abnormalities in the lung parenchyma. These considerations motivate the search for innovative lung image processing methods capable of assessing HRCT parameters [8] in a repeatable and quantitative manner. In this context, segmentation plays a crucial role. Although many professionals still prefer manual over automatic segmentation, two main drawbacks arise: (i) HRCT scans comprise hundreds of slices, making manual segmentation extremely time-intensive; and (ii) the result becomes highly operator-dependent. Since repeatability of the segmented result can only be guaranteed by computer-assisted methods [12], several automatic segmentation tools have been proposed [13,14,15,16,17]. In addition, automatic computer-based assessment may further improve the objectivity, sensitivity, and repeatability of such quantitative analysis (e.g., histogram analysis and texture patterns, see Figure 1). 

In this context, ‘radiomics’ has shown high potential for disease detection and treatment response prediction [18,19,20,21]. In contrast with traditional approaches, where HRCT images are inspected and subjectively interpreted, radiomics extracts a large set of quantitative features and analyses their statistical correlation with observable aspects of the disease (e.g., physiological parameters) to identify those of most relevance [22,23]. Nevertheless, radiomic results are highly influenced by the tissue region considered, and this becomes an important limitation when segmentation is performed manually. Radiomics provides reliable results only if the whole process is automatic and user-independent. Consequently, automatic segmentation becomes crucial to ensure reproducible prediction [24,25].

In this study, we investigate the correlation of HRCT features from thirty-two IPF patients with selected physiological parameters. Most advanced HRCT feature extraction algorithms based on texture analysis are computationally demanding. As a consequence, their application has remained confined to research studies. To avoid this computational burden, some studies have focused on HRCT histogram evaluation [23]. The latter requires considerably less computation and is therefore a viable option for clinical applications. Quantitative parameters based on image histograms have been used to evaluate lung fibrosis scores and, consequently, to monitor the extent and severity of IPF [26]. We emphasize that: (1) the pulmonary fibrosis fraction (PFF%) parameter shows significant negative correlation with both FVC and DLco [7], (2) the percentage of the volume of normally attenuated lung (NL%) closely correlates with lung physiology variables, showing a large area under the receiver operating characteristic curve (ROC) for detecting patients in moderate or advanced stages of IPF [8], (3) the high-attenuation area (HAA) parameter reflects physiological impairments correlating with physiological measures, with visual fibrosis scores [9], and with transplant-free survival [10]. Furthermore, HRCT histogram indices including kurtosis, skewness, mean lung density (MLD), median, and variance have been considered as well [11]. Among these, kurtosis has been identified as a strong predictor for mortality [9]. While they are extremely promising, these studies greatly differ in the type of software used to perform IPF analysis, and a consistent definition of the Hounsfield Unit (HU) range in which the whole lung volume should be measured is not discussed. HU is a dimensionless quantity obtained from a linear transformation of the attenuation coefficients measured in CT imaging, typically used to express voxel values in a standardized form. HU ranges from −1000 for air, to approximately 2000 for dense bone. Conventionally, the tissue with HU values between two thresholds is used to estimate lung volume. The lower threshold is typically set to −1024 HU, while the upper threshold is variable. The choice of the upper threshold has deep repercussions on the calculation of HRCT parameters and may affect the judgement of IPF severity. To understand the role of this threshold we investigated three different choices, namely −200, 0, and 200 HU as often encountered in the literature. To better motivate these choices, we review the IPF parameters introduced by several other researchers (from [7,8,9,10]):

- Salaffi et al. [7] proposed the PFF% parameter, defined as the percentage of the non-fibrotic area (from −1.024 HU to −700 HU) in the HRCT lung volume (from −1.024 HU to −200 HU). This motivates our −200 HU choice.
- Ohkubo et al. [8] proposed the NL% parameter, defined as the percentage of the normally attenuated lung (from −950 HU and −701 HU) in the HRCT lung volume (from −1.024 HU to 0 HU. This motivates our 0 HU choice.
- In Tanizawa et al. [9], HAAs and LAAs were defined as areas with attenuation values greater than −200 HU and less than −960 HU, respectively. HAA is indicative of parenchymal lesions, such as ground-glass opacity and reticulation, whereas LAA is indicative of emphysematous patches. Additionally, HAA% and LAA% were defined as percentages of the HRCT lung volume) occupied by HAA and LAA, respectively. Unfortunately, HU thresholds were not explicitly declared in this study.
- Ash et al. [10] introduced the HAA_A% parameter as an alternative to HAA. HAA_A% corresponds to the percentage of the HRCT lung volume that has a density from −250 to −600 HU. The HRCT lung volume was considered, in this case, from −1.024 HU to −200 HU.
- Finally, Klim et al. [11] obtained kurtosis, skewness, MLD, median, and variance values by choosing +200 HU as the upper threshold in the HRCT lung volume calculation. Of course, this motivated our last threshold choice.

In summary, we see that the −200, 0, and 200 HU thresholds are frequently encountered in the literature, and yet, the impact of these choices on the reliability of the IPF parameters has been largely overlooked. Therefore, not only did we investigate state-of-the-art HRCT parameters (listed in Table 1) to gain insight into their relationship with physiological parameters and identify potential surrogate measures from HRCT examinations in a real-life work setting, but we examined the effect of different HU threshold choices as well. 

A systematic statistical analysis among quantitative parameters obtained using different HU thresholds is essential to produce robust and accurate IPF biomarkers. Consequently, this study aims to extract and validate quantitative features from HRCT imaging and to create a “Medical Decision Support System” capable of improving routine clinical practice related to the diagnosis of IPF beyond the current standards (ATS/ERS/JRS/ALAT guidelines [4]). 

The paper is organized as follows. Section 2 describes the methodology to extract HRCT parameters. Section 3 describes the data used and illustrates the main results, while Section 4 is devoted to discussion.

## 2. Materials and Methods 

### 2.1. Patients

Our study evaluated retrospectively a set of thirty-two patients. All patients participated in a multidisciplinary team diagnosis of IPF as specified by the 2011 American Thoracic Society (ATS)/European Respiratory Society (ERS)/Japanese Respiratory Society (JRS)/Latin American Thoracic Association (LATA) IPF guidelines. Only patients with no-contrast and volumetric thin-section CT were included in the present study. All patients were treated, as recommended, with antifibrotic therapies (either pirfenidone or nintedanib). All clinical data were obtained from medical records. FVC and DLco were performed according to the ATS/ERS guidelines using Vmax Sensor Medics and Jaeger (VIASYS Healthcare; Yorba Linda, CA, USA), and the results were expressed as percentages of predicted values. Written informed consent was not obtained from the patients because of the retrospective nature of the study which used clinical and HRCT data accumulated in daily practice. Nevertheless, the study was approved by the ethical committee of the Policlinico-Vittorio Emanuele Hospital of Catania (.letter number 0039547, protocol QH-IPF, date 5 September 2018). 

### 2.2. High Resolution Computed Tomography (HRCT) Protocol

All patients underwent volumetric thin-section HRCT examinations using a CT Philips scanner (Philips Healthcare, Cleveland, OH, USA). Scans were obtained at full inspiration from the apex to the lung base with the patients in the supine position. Thin-section CT images (no more than 1.5 mm) were with sharp kernel imaging reconstruction, contiguous or overlapping images.

### 2.3. Quantitative HRCT Assessment

The quantitative analyses of HRCT datasets were performed using an in-house processing tool developed in MATLAB^®^ R2016a (The MathWorks, Natick, MA, USA), running on iMac (3.5 GHz Intel Core i7 processor, 16 GB memory random-access memory; Apple Computer, Cupertino, CA, USA) with Mac Operating System OS X El Capitan. First, to isolate the lungs from other tissues and structures, automatic HRCT segmentation was performed using a region growing algorithm [27] and isolating voxels between +200 and −1.024 HU. The same region growing method was used to eliminate the trachea. Next, a thoracic radiologist inspected the segmentation and, in the case of coarse anatomical misrecognition or inaccurate segmentation, manually guided the delineation. At this point, HRCT attenuation histograms were obtained. Proceeding further, we considered three different upper HU thresholds (−200 HU, 0 HU, and 200 HU) producing three derived HRTC histograms for each study. Finally, we calculated the values of the most common state-of-the-art HRCT parameters: PFF% [7], NL% [8], HAA%, LAA% [9], and HAA_A% [10], obtaining three different indices sets corresponding to each threshold. In addition to these parameters, other HRCT histogram indices including kurtosis, skewness, mean lung density (MLD), median, and variance were considered as well (Table 1). With concern to kurtosis, large values seem to indicate mild IPF, while low values may indicate severe IPF. Among the various formulas for calculating kurtosis, we used the formula which yields zero for a perfect normal distribution:

(1) $$Kurtosis=\frac{{\left(x-y\right)}^{4}}{{\left[\sum {\left(x-y\right)}^{2}\right]}^{2}}-3$$

where x indicates density (ranging from −1024 to the selected upper HU threshold) and y is the mean attenuation value from the histogram [11]. Skewness describes the degree of asymmetry of a histogram, with a long right tail indicating positive skewness [11]. Table 1 presents a complete list of the HRCT parameters considered in our analysis.

### 2.4. Statistical Analysis

Statistical analyses were carried out using the computing environment R (R Development Core Team, 2005). Characteristics of the study population were expressed using mean and standard deviation (SD). FVC and DLco indices were put in relationship with HRCT measures. Their relationship was examined using several regression models (general linear model (GLM), generalized linear mixed model (GLMM), and vector generalized linear models (VGLM)) checking for potential differences at different thresholds for the upper limit of the whole lung (−200, 0, and +200 HU). A p-value less than 0.05 was considered significant. 

## 3. Results

### 3.1. Subsection

**Figure 2:**
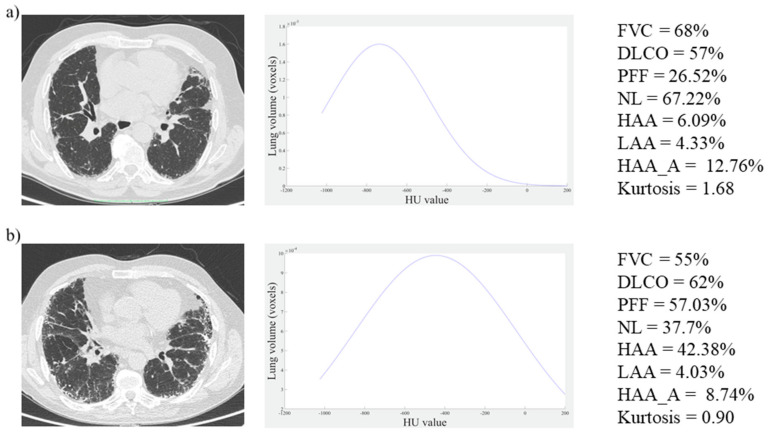
Two representative clinical cases: HRCT images (left), HRCT lung histograms (centre), pulmonary function tests and quantitative parameters (right): (**a**) Patient affected by moderate idiopathic pulmonary fibrosis (IPF), (**b**) Patient with more severe evidence of IPF. Forced vital capacity (FVC), diffusion capacity for carbon monoxide (DLCO), PFF, NL, HAA, LAA, and HAA_A parameters are expressed in percentage values while kurtosis is expressed in absolute value.

Patient’s ages ranged from 55 to 82 years (age mean ± SD, 68.34 ± 6.47), and 6 out of 32 patients were women. FVC and DLco were 89.03 ± 19.63% and 62.84 ± 17.06%, respectively. HRCT parameters were calculated considering the three different upper HU thresholds (−200, 0, and +200). Representative images from patients with less severe (patient 1) and more severe (patient 2) evidence of IPF are shown in Figure 2. Histograms of distribution, pulmonary function tests, and some HRCT parameters are also reported for each subject considering HU < 200. 

### 3.2. Exploratory Analysis

**Figure 3:**
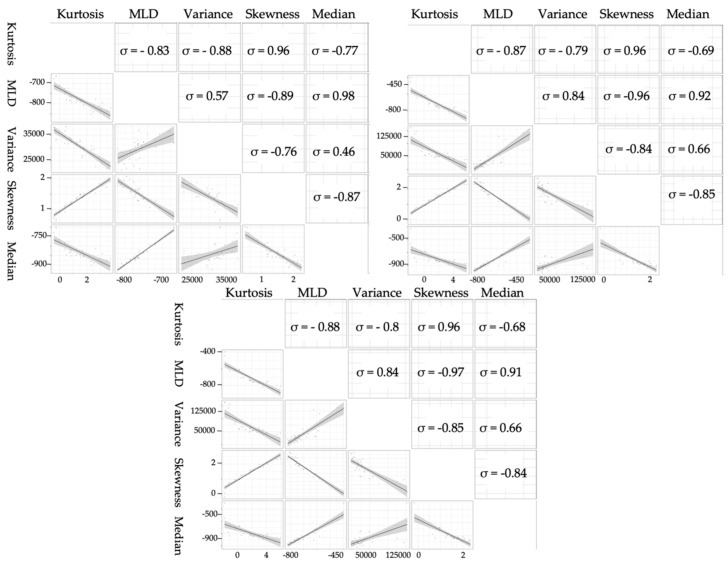
Pairwise scatter plot matrix, and correlation coefficients (ρ) of HRCT histogram indices including kurtosis, mean lung density (MLD), variance, skewness, and median at -200 (left), 0 (right), and 200 HU (centre).

Starting with an exploratory analysis of HRCT measures, the correlation between these measures was computed. Statistical synthesis measures of density (kurtosis, skewness, MLD, median, and variance) [11] were highly correlated at each threshold, as shown in Figure 3. As a result, we only considered kurtosis, given that it is the most useful measure in assessing patient condition, as reported in [9]. 

Kurtosis, PFF, NL, HAA LAA, and HAA_A of the 32 patients are summarized in Table 2 considering the three different upper HU thresholds (-200, 0, and +200). 

**Figure 4:**
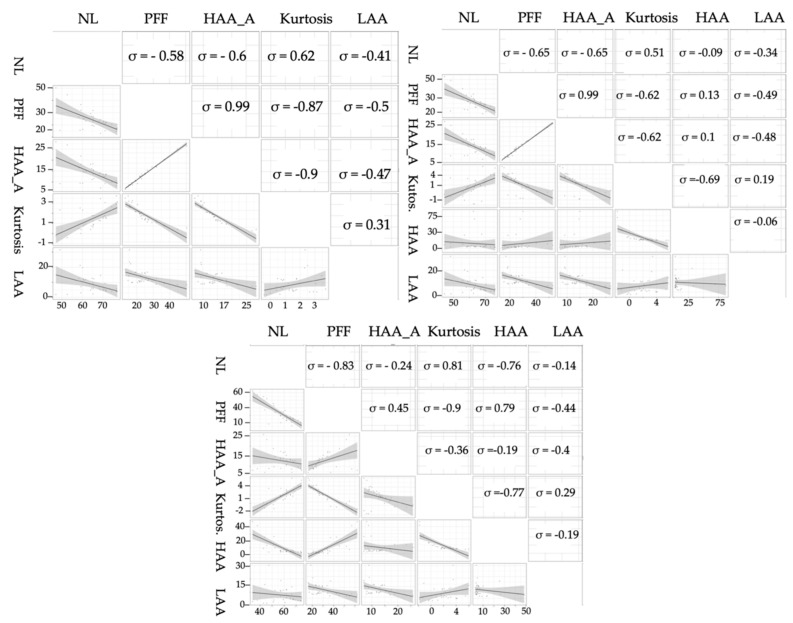
Pairwise scatter plot matrix, and correlation coefficients (ρ) of HRCT histogram indices including NL, PFF, HAA_A, kurtosis, HAA, LAA at −200 (left), 0 (right), and 200 (centre) HU. HAA, being defined as the areas with attenuation values greater than −200 HU, is not shown in the first matrix. In NL, PFF, HAA_A, HAA, LAA parameters, the ‘%’ has been omitted for ease of reading.

Their correlation varied consistently depending on the threshold, as shown in Figure 4 and further highlighted in Table 3 and Table 4. HAA, defined as the areas with attenuation values greater than −200 HU, is not shown in Table 3 for the −200 HU threshold. Minimal differences between measure at −200 or 0 HU were identified, while at +200 HU the magnitude of the relationships changes noticeably.

### 3.3. Models Analysis

As complex models are often hindered by convergence issues, we leveraged a Poisson model approximation to evaluate the relationship between the spirometry tests (FVC, DLco) and the HRCT measures. Poisson models were fitted over data using the score given from the Gender-Age-Physiology (GAP) index [28] as the response variable, and each one of the HRCT measures at different thresholds as the explanatory variable. GAP provides a screening method to determine the average risk of mortality of patients with IPF stratifying them into three stages based on clinical (sex and age) and physiological (FVC and DLco) variables. It provides 1-, 2-, and 3-year mortality estimates. GAP stage 3 indicates the worst-case scenario. 

Table 5 highlights how the NL measure is most strongly related to the response (*p* = 0.009). In particular, it has at least one more point of log-likelihood (minimum value to affirm that two models do not contain the same information) beyond the second-best measure, which shows a borderline *p*-value (0.047). Measures at 0 or -200 HU show no difference (correlation coefficient = 0.99), while measures at +200 HU seem to hold less information, although some measures are more robust than others to threshold choices.

Mixed models, using FVC and DLco as response variables, were considered for the NL measure at −200 and +200 HU. A gamma distribution was assumed since FVC and DLco are both positive and continuous (logarithmic link for convergence problems). As shown in Table 6, NL seems to have a strong relationship with both responses whatever the threshold (*p*-value < 0.01).

## 4. Discussion

In recent years, many studies have focused on recognizing diagnostic indicators for complex diseases such as IPF. HRCT examinations present crucial advantages for the management of IPF patients, both because they provide increased accuracy and because they reduce the need for a lung biopsy. The non-invasiveness of HRCT studies, the large amount of data they provide, and the set of parameters, correlated to the disease that can be derived from such data, make HRCT a suitable tool for IPF studies. Unfortunately, most of the currently published studies are based only on qualitative evaluations of HRCT, with the drawback that visual analysis is time-consuming and therefore of limited use in clinical environments. Although several analysis methods have been proposed to objectively evaluate HRCT examinations, it is still not entirely clear which index should be adopted. Recent studies investigating histogram-based indices for IPF evaluations provided very promising results. Nevertheless, such investigations widely differ in terms of the type of software used to perform the analysis, and lack a standard definition of the HU range in which the whole lung volume should be measured. The major source of uncertainty is the upper HU threshold. 

We leveraged clinically acquired volumetric data in our study to investigate whether some correlation exists between densitometric HRCT measures and pulmonary functional tests. Additionally, as the choice of a proper upper HU threshold is a key source of uncertainty which has been poorly investigated in the literature, we explored the variability of these indices for different choices of HU ranges for parenchyma measurement. To date, choosing the upper HU threshold has been largely subjective rather than driven by true insight. Therefore, we focused on the three values which seem to be the most common options in the scientific literature (−200 HU, 0 HU, and +200 HU). For this reason, this paper addresses the HU threshold issue in quantifying HRCT indices, which must be understood to make them fully reliable and to set a proper standard in the field. Our analysis demonstrated relevant differences in the results by the three thresholds. Using the GAP index as the response variable and the HRCT measures at the three different thresholds as explanatory variables, we discovered that the best diagnostic performance is achieved for the −200 HU threshold, with the most significant variables being Kurtosis and NL (*p* = 0.049 and *p* = 0.009, respectively). 

The need to identify the best diagnostic performance is related to the increasing use of quantitative indices as biomarkers for monitoring disease behaviour. This biological behaviour needs to be defined not only on pulmonary function tests, but also on functional parameters obtained by a kind of radiomics analysis. Therefore, we sought to assess the correct method for quantitative analysis by exploring the relationship between pulmonary function test measurements and HRCT indices with a Poisson model approximation to identify the measurements of highest log-likelihood value. This relationship is very important in the management of diseases; some progressive patterns of disease, which have been now labelled as “progressive phenotypes of fibrosis”, will be monitored by evaluating even very small variations of these functional and quantitative parameters. Consequently, the measurement of quantitative features extracted from HRCT images needs to be more accurate, and the methodology becomes relevant. Optimal performance is needed to assess diagnosis and to determine management.

This study, however, has some limitations. For example, this kind of study may be influenced by the patient inspiration level. Additionally, an incorrect manoeuvre or the presence of artefacts may affect the extraction of quantitative parameters from HRCT examinations. Spirometric control was not used at the time of data collection. As such, given the retrospective nature of the study, we cannot ensure that inspiration was optimal during acquisition. Nevertheless, the quality of all HRCT studies was reviewed and approved by our radiologists before their inclusion within the present study. Further, our results were obtained from a relatively small number of patients, and validation on a larger sample would be advisable. Despite these limitations, our study showed a stronger relationship between NL and both the FVC and DLco parameters compared to the other HRCT measures considered. This suggests the usefulness of NL in association with pulmonary function test values as a diagnostic marker for disease behaviour. Nonetheless, this result could be affected by a low specificity (high false-positive rate) if applied blindly in a general lung disease patient group. For this reason, it is important to stress the need for further studies to evaluate its ability to predict patient survival and to better understand if it might also rival spirometry tests in terms of diagnostic performance. Specifically, a prognosis evaluation with a sufficient time interval to assess longitudinal disease behaviour will be reserved for an upcoming paper by comparing state-of-the-art HRCT features with transplant-free survival and with the variation of spirometry measures over time. Finally, because IPF affects males more frequently (ratio 2:1), the majority of patients in our study were male. Obviously, different genders exhibit different thoracic dimensions and configurations (for example, the lung region, ribcage dimension, and diaphragm length are comparatively smaller in females). In the future, we will systematically investigate the impact of such factors.

## 5. Conclusions

Based on our results, computer-based analysis provides a promising tool for the assessment of patients with IPF disease. In particular, among all the parameters we considered in the quantification of HRCT images, NL at −200 HU demonstrated the strongest correlation with disease severity. It can be leveraged for IPF diagnosis and to aid decision making in daily clinical practice. Studies featuring a larger patient population are advised to further confirm the present findings.

## Figures and Tables

**Table 1:** State-of-the-art-parameters obtained from HRCT found in recent literature. In the specific, high-attenuation area (HAA)% [9] is defined as areas with attenuation values greater than −200 Hounsfield Unit (HU). HAA_A% [10] is defined as the percentage of the whole lung volume with a density between −250 and −600 HU. Pulmonary fibrosis fraction (PFF), normally attenuated lung (NL), HAA, LAA, and HAA parameters are expressed in percentage values (%) while kurtosis, skewness, mean lung density, median, and variance are expressed in absolute values.

HRCT Parameter	Acronyms	Reference
Pulmonary fibrosis fraction	PFF%	[7]
Normally Attenuated Lung	NL%	[8]
High Attenuation Area	HAA%	[9]
Low Attenuation Area	LAA%	[9]
High Attenuation Area	HAA_A%	[10]
Kurtosis	-	[11]
Skewness	-	[11]
Mean Lung Density	MLD	[11]
Median	-	[11]
Variance	-	[11]

**Table 2:** Mean (%) ± Standard Deviation (SD) and median values (%) of HRCT parameters obtained considering the three different HU thresholds. HAA, defined as the areas with attenuation values greater than −200 HU, is not shown in the first column.

	−200 HU		0 HU		200 HU	
Parameters	Mean ± SD	Median	Mean ± SD	Median	Mean ± SD	Median
NL%	65.12 ± 7.41	65.24	61.51 ± 6.68	62.96	58.99 ± 10.64	61.29
PFF%	23.98 ± 8.25	22.71	26.84 ± 9.32	24.80	31.05 ± 11.94	26.40
HAA%	n.a.	n.a.	14.84 ± 24.97	3.95	9.42 ± 10.99	4.88
LAA%	8.51 ± 6.42	5.95	9.15 ± 5.50	6.95	7.19 ± 4.59	5.57
HAA_A%	14.09 ± 4.87	13.61	14.16 ± 4.74	13.81	12.70 ± 4.40	12.23
Kurtosis	1.49 ± 1.08	1.39	1.65 ± 3.02	1.69	1.91 ± 3.33	1.95

n.a.= not applicable

**Table 3:** Correlation matrix of HRCT histogram indices including NL, PFF, HAA_A, kurtosis, and LAA at −200 HU threshold. HAA, defined as the areas with attenuation values greater than −200 HU, is not shown. In NL, PFF, HAA_A, HAA, LAA parameters, the ‘%’ has been omitted for ease of reading.

	HU: −200				
	NL	PFF	HAA_A	Kurtosis	LAA
NL	1.00	−0.58	−0.60	0.62	−0.41
PFF	−0.58	1.00	0.99	−0.87	−0.50
HAA_A	−0.60	0.99	1.00	−0.90	−0.47
Kurtosis	0.62	−0.87	−0.90	1.00	0.31
LAA	−0.41	−0.50	−0.47	0.31	1

**Table 4:** Correlation matrix of HRCT histogram indices including NL, PFF, HAA_A, kurtosis, HAA and LAA at 0 and +200 HU threshold. In NL, PFF, HAA_A, HAA, LAA parameters, the ‘%’ has been omitted for ease of reading.

	HU: 0						HU: 200					
	NL	PFF	HAA_A	Kurtosis	HAA	LAA	NL	PFF	HAA_A	Kurtosis	HAA	LAA
NL	1.00	−0.65	−0.65	0.51	−0.10	−0.34	1.00	−0.83	−0.24	0.81	−0.76	−0.14
PFF	−0.65	1.00	0.99	−0.62	0.13	−0.49	−0.83	1.00	0.45	−0.90	0.79	−0.44
HAA_A	−0.65	0.99	1.00	−0.62	0.10	−0.48	−0.24	0.45	1.00	−0.36	−0.19	−0.40
Kurtosis	0.51	−0.62	−0.62	1.00	−0.69	1.00	0.81	−0.90	−0.36	1.00	−0.77	0.29
HAA	−0.10	0.13	0.10	−0.69	1.00	−0.06	−0.76	0.79	−0.19	−0.77	1.00	−0.19
LAA	−0.34	−0.49	−0.48	0.19	−0.06	1.00	−0.14	−0.44	−0.40	0.29	−0.19	1.00

**Table 5:** Poisson models were fitted over HRCT data, using the Gender-Age-Physiology (GAP) index as a response variable, and each of the HRCT measures at different thresholds as explanatory variables.

	200 HU		0 HU		-200 HU	
	*p*-Value	Log-lik	*p*-Value	Log-lik	*p*-Value	Log-lik
Kurtosis	0.28	−33.40	0.06	−31.97	0.047	−31.80
NL%	0.08	−32.46	0.008	−30.68	0.009	−30.76
PFF%	0.34	−33.54	0.12	−32.89	0.13	−32.94
HAA_A%	0.10	−32.71	0.09	−32.66	0.09	−32.68
HAA%	0.98	−33.96	0.85	−33.95	n.a.	n.a.
LAA%	0.19	−33.22	0.24	−33.36	0.24	−33.36

n.a.= not applicable

**Table 6:** Mixed models using FVC and DLco as response variables, and NL at −200 HU and +200 HU as the explanatory variable. The regression coefficient estimates (β) and their standard error (SE) are reported on the link function scale (natural logarithm -Ln-). For immediate reading, coefficient estimates were exponentiated (Exp). Finally, the comparison was carried out in terms of log-likelihood (Log-lik).

	200 HU					−200 HU				
	β;	Exp (β)	SE [β]	*p*-Value	Log-lik	β	Exp (β)	SE [β]	*p*-Value	Log-lik
Ln (FVC)	0.006	1.006	0.002	7.38 × 10^−4^	−233	0.01	1.003	0.003	1.44 × 10^−3^	−234
Ln (DLco)	0.007	1.007	0.003	8.61 × 10^−3^	−230	0.002	1.003	0.005	1.45 × 10^−4^	−227
